# Association between lifestyle behaviors and health-related quality of life among primary health care physicians in China: A cross-sectional study

**DOI:** 10.3389/fpubh.2023.1131031

**Published:** 2023-03-08

**Authors:** Yisha Lin, Yuankai Huang, Xiaoyu Xi

**Affiliations:** The Research Center of National Drug Policy & Ecosystem, China Pharmaceutical University, Nanjing, Jiangsu, China

**Keywords:** health-related quality of life (HRQoL), lifestyle, primary health care, physician, China

## Abstract

**Background:**

Primary health care (PHC) serves as the gatekeeper of health system and PHC physicians take on significant obligations to provide health care services in the pursuit of Universal Health Coverage (UHC). PHC physicians' health-related quality of life (HRQoL) can have a strong impact on patients, physicians and the health care system. Lifestyle interventions are found to be effective to improve HRQoL. The purpose of this study was to evaluate the association between lifestyle behaviors and HRQoL among PHC physicians, so that lifestyle intervention can be tailored by policy makers for health promotion.

**Methods:**

A survey covering 31 provinces and administrative regions in China was conducted in 2020 using a stratified sampling strategy. Data on sociodemographic characteristics lifestyle behaviors and HRQoL were collected by a self-administered questionnaire. HRQoL was measured through EuroQol-five dimension-five level (EQ-5D-5L) instrument. A Tobit regression model was performed to evaluate the association between sociodemographic characteristics, lifestyle behaviors and HRQoL.

**Results:**

Among 894 PHC physicians who completed the survey, Anxiety/Depression (AD) was the dimension with the most problems reported (18.1%). Regular daily routine (β = 0.025, 95%CI 0.004 to 0.045) and good sleep quality (β = 0.049, 95% CI = 0.029 to 0.069) were protective factors for HRQoL, while smoking (β = −0.027, 95% CI = −0.079 to −0.003) and frequency of eating breakfast (β = −0.041, 95%CI = −0.079 to −0.003) were negatively associated with HRQoL. Physical activity and alcohol drinking were not significantly associated with HRQoL.

**Conclusion:**

These findings suggest that tailored interventions on daily routine, improving sleep quality, and tobacco control among PHC physicians may be effective strategies to improve their HRQoL.

## 1. Introduction

Primary health care (PHC) is defined as a whole-of-society approach to effectively organizing and strengthening national health systems to bring services for health and well-being closer to communities, from health promotion to disease prevention, treatment, rehabilitation, palliative care and more (1). PHC serves as the gatekeeper of health system (2) and provides the foundation for the strengthening of the essential public health functions to confront public health crises such as COVID-19. Physicians, the backbone of the primary health care workforce (3), play a vital role in coordinating a person's care needs, from prevention to disease management to curative care (4). With the goal of Universal Health Coverage (UHC) and strengthening PHC system (5), primary health care physicians are expected to take on a heavier burden to provide health care services, particularly in times of crisis such as the COVID-19 pandemic. A number of studies have demonstrated a high prevalence of physical and mental illness, job burnout (6, 7), sleep disturbance (8) and even suicide (9) among physicians, concerning unsatisfactory health-related quality of life (HRQoL).

HRQoL is a multidimensional concept representing both positive and negative aspects of physical and psychological health, social functioning, and overall well-being (10). Poor HRQoL of physicians could have a negative impact on work performance and patient outcomes in addition to their individual health (11, 12). As a result, addressing HRQoL of physicians benefits patients, physicians and the health care system. In China, the total number of PHC physicians was 1.246 million and PHC institutions provided 50.2% of outpatient care (4.3 billion visits) and 14.5% of inpatient care (35.9 million hospital admissions) in 2021 (13). Given the great amount of health care service provided by PHC physicians, it is an urgent public health issue that HRQoL of PHC physicians should be improved in the light of enhancing PHC services and physicians' performance.

It is evidently identified that lifestyle behaviors affect people's health, HRQoL and life expectancy (14–17). Moreover, lifestyle interventions were found to be effective to improve HRQoL (18, 19) as well as the implementation of lifestyle interventions in the workplace has been proven to be cost-effective for both employers and society (20, 21). The occupational category should be considered when designing workplace health promotion programs in the belief that the occupational category produces significant differences in lifestyle behaviors (22–24). In addition, the association between lifestyle behaviors and HRQoL varies between occupations. For instance, smoking was not found to be associated with HRQoL among government employees (25) while current smoker had lower HRQoL in professional drivers (26). The result of a study suggested that breakfast, exercise, smoking and drinking should be given priority to health promotion at work for doctors (23). But it is unknown whether these lifestyle behaviors are risky or not for HRQoL of PHC physicians. Physicians are considered as a group with a higher level of health literacy (27), and their better perceived capacities in finding, understanding and applying health information could directly lead to better physical health and subjective well-being (28). For example, healthcare professionals are more likely to attend medical programs due to their work environment. A research found that smoking and alcohol consumption were not related to quality of life as anticipated among residents participating in the medical checkups (29). Considering the above, the influence of lifestyle behaviors on HRQoL of physicians may not be the same as that in the general population or other occupations. Moreover, despite knowledge of the significance of healthy lifestyle behaviors, healthcare workers do not adopt healthy lifestyle behaviors for various reasons (30–33), such as a false feeling of “protection” due to their medical knowledge, excessive workload, lack of time or motivation, and the tendency to prioritize their patients' health over their own. Knowledge of the relationship between lifestyle behaviors and HRQoL among physicians would help tailor more effective health-promoting interventions.

Prior work has documented the relationship between lifestyle behaviors and HRQoL in different population. However, these studies have primarily focused on patients with different diseases (14, 18, 34–36) and general populations including adolescents, adults and the elderly from different countries or regions (17, 20, 21). In research to date, few studies have examined such relationships in healthcare professional groups (37), especially little is known about that in primary health care physicians. A study investigated the relationship between lifestyle factors (smoking, BMI, cooking oil, meals out per week, total fruit and vegetable intake per day, physical activity level, and hours of TV, laptop, or internet use per day) and quality of life among 72 PHC physicians in Saudi Arabia (38) with limitation of small sample size. However, other important lifestyle behaviors' influence [e.g., alcohol consumption, sleep quality (16, 39–41)] on HRQoL of PHC physicians has not been properly studied yet. Combined consideration of both occupational characteristics of healthcare professionals (23, 30, 42) [e.g. atypical work schedules (43), heavy workload and stress (44)] and lifestyle behaviors which have been identified as potential factors affecting HRQoL in the Chinese population (39, 45–47), lifestyle behaviors including daily routine, breakfast, sleep quality, smoking, drinking and physical activity should be taken into account. The absence of relative research on the relationship between such lifestyle behaviors and HRQoL among PHC physicians, however, is an impediment to health policy consideration for improving their HRQoL.

The aim of this study was to evaluate the association between lifestyle behaviors and HRQoL among Chinese primary health care physicians. Such important information could be informative for local health care policy makers and researchers to consider at which levels effective lifestyle interventions should be implemented to improve the HRQoL of primary health care physicians.

## 2. Methods

### 2.1. Setting and study design

This cross-sectional study covering 31 provinces and administrative regions in China in 2020 was designed to investigate the associations between some lifestyle behaviors and HRQoL. PHC physicians were recruited and data were collected by a self-administered questionnaire. The inclusion criteria of respondents were: 18 years or older, able to fill in the questionnaire independently and employed in primary health care sectors as physicians in China. A stratified sampling strategy was adopted, and the detailed steps were as follows: (1) The sample of the study included 31 provinces and administrative regions in China. The cities or districts of each region were categorized into three groups (high, medium, and low) according to GDP per capita in 2018, forming a total of 93 groups. (2) At least two local PHC institutions including community health centers, township health centers, and community health stations were selected respectively for convenience sampling in each group, forming a total of 558 samples of PHC institution. (3) In each PHC institution, at least two physicians selected by convenience sampling were willing to participate in and complete the investigation.

### 2.2. Instruments

#### 2.2.1. Sociodemographic information

Sociodemographic information was collected including age, sex, height, weight, marital status, number of children, caregiving status, annual household income, educational level, job title, type of residence and chronic diseases. The sociodemographic characteristics mentioned above were included in the study as covariate. Age was divided into three groups: young adults (age 18–44 years), middle-aged adults (age 45–59 years), and older adults (age over 60 years) (48). Body mass index (BMI) was computed from self-reported weight and height and was divided into three groups: BMI < 18.5, 18.5 ≤ BMI ≤ 24.9, BMI ≥ 25 (49). Annual household income was categorized into three levels (RMB): < ¥80,000, ¥80,000 ~ ¥1,50,000 and >¥1,50,000.

#### 2.2.2. Lifestyle behaviors profile

The following health-related lifestyle behaviors were included in the study as independent variables: daily routine, sleep quality, breakfast, smoking, drinking and physical activity. The item “daily routine” was referred to as the established patterns of waking, eating, sleeping, and organizing one's time daily (50), which was evaluated into two statuses: irregular and regular. Sleep quality was answered by “not good” and “good” subjectively. Frequency of eating breakfast weekly means how many times individuals had breakfast in 1 week in the last 6 months, which was divided into two groups: < 4 times and at least 4 times. Both smoking and drinking status were categorized by yes or no. Smoking was defined as at least one cigarette per day in the last 6 months and drinking was defined as at least once a week in the last 6 months. Physical activity was classified as to whether the respondent practice any sports, exercise or other physical activity for at least 30 min during a usual week.

#### 2.2.3. Measurement of HRQoL

HRQoL was measured by the Chinese version of the EuroQol-five dimension-five level (EQ-5D-5L), consisting of a descriptive system and EQ- 5D visual analog scale (EQ-VAS) (51). EQ-5D-5L is one of the major self-reported instruments to evaluate HRQoL due to its simplicity, low respondent burden and high universal acceptance (52). It includes five dimensions: Mobility (MO), Self-Care (SC), Usual Activities (UA), Pain/Discomfort (PD), and Anxiety/Depression (AD). Each dimension has five levels. The responses for the five dimensions can be combined in a 5-digit number describing the respondent's health state (from 11111 meaning no problems at all to 55555 meaning extreme problems in all five dimensions). Then the responses obtained were converted to the EQ-5D utility index based on Chinese value sets (53) to represent HRQoL.

#### 2.2.4. Questionnaire validation

The validity, rationality, comprehensibility, and readability of the questionnaire had been verified by experts and the results of a pilot survey in community health centers in Nanjing, Jiangsu province, China. Based on the feedback from the pilot survey, the research team revised the questionnaire and formulate the final version.

### 2.3. Data collection

A total of 500 undergraduate students majoring in clinical pharmacy or pharmacy were recruited as investigators. In order to ensure the survey quality, each investigator was strictly trained before the investigation, including understanding the principles and methods of survey design, and standardizing the definition. In the process of collecting data, investigators provided the participants with an electronic device—Interview Master, a survey app in WeChat, and gave them instructions about how to complete the questionnaire on the app. Then the responses were automatically converted into electronic data for analysis software. A total of 5 master students were recruited and trained as auditors. If auditors found obvious errors in the data, the data would be returned to the data collectors and then they would verify it with the respondents.

### 2.4. Data analysis

Data were analyzed by STATA 17 for Windows and IBM SPSS Statistics 26 for Windows. Means and standard deviations (SD) were used to describe continuous variable data as well as frequencies and percentages are used to describe categorical variable data. Differences in HRQoL based on categories of sociodemographic characteristics and lifestyle behaviors were explored using Mann–Whitney U (two groups) and Kruskal–Wallis one-way analysis of variance (multiple groups) due to the abnormal distribution of the EQ-5D utility index. The EQ-5D utility index was skewed and it was censored at 1 (54), so a Tobit regression model was chosen to explore the potential effects of sociodemographic characteristics and lifestyle behaviors on HRQoL. The level of statistical significance was set at *p* < 0.05 in all analyses. Variance inflation factor (VIF) was used to test the multicollinearity. Robust test was conducted by changing conversion formula to calculate HRQoL. The formula was as follows: 1 subtract the sum of the five dimensions scores divided by 25. The value represented HRQoL by a single index ranging from 0 (for 55555) to 0.8 (for 11111).


$$\begin{array}{l} \text{HRQoL}=1-\left(\text{MO}+\text{SC}+\text{UA}+\text{PD}+\text{AD}\right)÷25 \end{array}$$

## 3. Results

### 3.1. Sociodemographic and lifestyle behaviors

A total of 1,227 participants answered the questionnaire, of which 894 (73%) provided valid information on all included variables (e.g., the measures of weight and height both were valid, current health status was consistent with chronic disease prevalence). Table 1 summarizes the sociodemographic characteristics and lifestyle behaviors of the participants. A total of 894 participants were included and had an average HRQoL of 0.978 (SD = 0.045). The mean age was 41.19 (SD = 9.15), among which the ratio of male to female respondents was around1: 1.05. Annual household income of respondents was ¥1,62,600 (SD = 1,15,500) on average. Majority of them were married (89.60%) and had at least one child (87.47%). Approximately 90.00% of participants reported not having any chronic diseases diagnosed in the hospital at the time of completing the survey. For their lifestyle behaviors, over half of them had regular daily routine (67.67%) and good sleep quality (58.17%). Almost all of them had breakfast at least 4 times a week (92.28%). Most of them did not smoke (78.97%) or consume alcohol (55.26%). The percentage of respondents who never participated in physical activity was 9.06%.

**Table 1:** Sociodemographic characteristics, lifestyle behaviors, and HRQoL.

**Characteristics**	***n* (%)**	**Mean (SD)**	***p-*value**
All	894 (100.00)	0.978 ± 0.045	
Sex			0.795
Male	458 (51.23)	0.976 ± 0.047	
Female	436 (48.77)	0.979 ± 0.043	
Age			0.023^*^
< 45	558 (62.42)	0.982 ± 0.036	
45 ~ 59	312 (34.9)	0.971 ± 0.056	
≥60	24 (2.68)	0.953 ± 0.061	
BMI			0.014^*^
< 18.5	53 (5.93)	0.988 ± 0.028	
18.5 ~ 24.9	686 (76.73)	0.979 ± 0.044	
≥25	155 (17.34)	0.966 ± 0.052	
Education			0.992
Below undergraduate	330 (36.91)	0.976 ± 0.049	
Undergraduate	458 (51.23)	0.978 ± 0.043	
Above undergraduate	106 (11.86)	0.978 ± 0.045	
Type of residence			0.076
Urban	579 (64.77)	0.979 ± 0.045	
Rural	315 (35.23)	0.975 ± 0.045	
Marital status			0.085
Single	84 (9.4)	0.986 ± 0.035	
Married	801 (89.6)	0.977 ± 0.046	
Others (e.g., Divorced)	9 (1.01)	0.952 ± 0.060	
Annual household income			0.016^*^
<¥80,000	151 (16.89)	0.989 ± 0.030	
¥80,000 ~¥1,50,000	313 (35.01)	0.976 ± 0.044	
>¥1,50,000	430 (48.1)	0.975 ± 0.050	
Number of children			0.301
0	112 (12.53)	0.984 ± 0.036	
1 ~ 2	516 (57.72)	0.976 ± 0.049	
>2	266 (29.75)	0.978 ± 0.041	
Caregiving status			0.300
Not caring for the elderly	642 (71.81)	0.978 ± 0.046	
Care for the elderly	252 (28.19)	0.976 ± 0.042	
Title			0.288
Below middle	393 (43.96)	0.981 ± 0.039	
Middle	383 (42.84)	0.976 ± 0.050	
Above middle	118 (13.2)	0.972 ± 0.049	
Enrolment			0.153
Contract	331 (37.02)	0.977 ± 0.041	
Permanent	563 (62.98)	0.978 ± 0.048	
Commercial insurance			0.921
No	561 (62.75)	0.978 ± 0.045	
Yes	333 (37.25)	0.977 ± 0.045	
Chronic diseases			< 0.001^**^
No	804 (89.93)	0.983 ± 0.036	
Yes	90 (10.07)	0.925 ± 0.078	
Daily routine			< 0.001^**^
Irregular	289 (32.33)	0.971 ± 0.046	
Regular	605 (67.67)	0.981 ± 0.045	
Sleep quality			< 0.001^**^
Not good	374 (41.83)	0.968 ± 0.003	
Good	520 (58.17)	0.985 ± 0.002	
Breakfast			0.290
< 4 times/week	69 (7.72)	0.982 ± 0.041	
≥4 times/week	825 (92.28)	0.977 ± 0.046	
Smoking			0.054
No	706 (78.97)	0.979 ± 0.042	
Yes	188 (21.03)	0.971 ± 0.054	
Drinking			0.095
No	494 (55.26)	0.981 ± 0.041	
Yes	400 (44.74)	0.974 ± 0.050	
Physical activity			0.013^*^
No	81 (9.06)	0.964 ± 0.057	
Yes	813 (90.94)	0.979 ± 0.044	

* p < 0.05 and

** p < 0.01, respectively.

### 3.2. EQ-5D-5L dimensional profile and HRQoL

Five levels on the EQ-5D dimension included no problem, slight, moderate, severe, and extreme problems. Frequency of item response in each EQ-5D-5L dimension as follows: MO (97.8%, 2.0%, 0.2%, 0.0%, 0.0%), SC (99.6%, 0.4%, 0.0%, 0.0%, 0.0%), UA (98.2%, 1.8%, 0.0%, 0.0%, 0.0%), PD (83.2%, 16.1%, 0.7%, 0.0%, 0.0%) and AD (81.9%, 17.2%, 0.9%, 0.0%, 0.0%). In this survey, the largest number of participants reported problems in the AD dimension (18.1%), followed by the PD dimension (16.8%). Roughly 99% of participants had no problem in the other three dimensions. The least problematic dimension was the SC dimension accounting for only 0.4%.

The results of HRQoL for each factor were also shown in Table 1. Significant differences were found in the subgroups of age, BMI, and annual household income. Younger group (age < 45) had better HRQoL (0.982 ± 0.036) than the older group (0.971 ± 0.056, 0.953 ± 0.061 respectively). Highest BMI group (BMI ≥ 25) had the lowest HRQoL (0.966 ± 0.052), whereas lowest BMI group (BMI < 18.5) had the highest HRQoL (0.988 ± 0.028). HRQoL was progressively lower with an increase in income categories (*p* = 0.016), and lowest in those with an annual income over ¥1,50,000 (0.975 ± 0.050). Participants suffering from chronic diseases had lower HRQoL (0.925 ± 0.078) than those without chronic disease (0.978 ± 0.045). Regarding lifestyle behaviors, significant differences were found in some factors: daily routine (*p* < 0.001), sleep quality (*p* < 0.001) and physical activity (*p* = 0.013). In the case of daily routine, the average HRQoL of participants who answered “not regular” was 0.971 ± 0.046, while that of participants who answered “regular” was 0.981 ± 0.045. Participants who had good sleep quality and participated in physical activity had better HRQoL. Differences in HRQoL based on other lifestyle behaviors (e.g., breakfast, smoking and drinking) did not reach a statistically significant level.

### 3.3. Regression analysis

The Tobit regression analysis revealed that several demographic characteristics and lifestyle behaviors were significantly associated with HRQoL in the adjusted model (Pseudo *R*^2^ = 0.6038) (Table 2). Female (β = −0.029, *p* = 0.014, 95% CI = −0.052 to −0.006) and higher annual household income (β = −0.052, *p* = 0.001, 95% CI −0.083 to −0.021 and β = −0.050, *p* = 0.002, 95% CI = −0.082 to −0.019) were a risk factor for HRQoL among participants. Suffering from chronic diseases (β = −0.118, *p* < 0.001, 95% CI = −0.146 to −0.090) and rural residence (β = −0.028, *p* = 0.011, 95% CI = −0.049 to −0.006) were negatively associated with HRQoL of respondents. In addition, a negative correlation was identified between HRQoL and the frequency of eating breakfast (β = −0.041, *p* = 0.036, 95% CI = −0.079 to −0.003). A positive association was observed between HRQoL and regular daily routine (β = 0.025, *p* = 0.018, 95% CI = 0.004 to 0.045) as well as good sleep quality (β = 0.049, *p* < 0.001, 95% CI = 0.029 to 0.069). Smoking status was significantly associated with the HRQoL. Compared with non-smokers, smokers report significantly lower HRQoL (β = −0.027, *p* = 0.043, 95% CI = −0.079 to −0.003). Associations between other lifestyle behaviors (drinking and physical activity) and HRQoL did not reach a statistically significant level. Variance inflation factor (VIF) for each independent variable was well below the recommended threshold of 10 (55), suggesting that the models did not have a multicollinearity issue. Changing conversion formula to calculate HRQoL did not materially alter the conclusions of the original tobit model (Appendix Table 1). Associations between sleep quality (β = 0.059, *p* < 0.001, 95% CI = 0.037 to 0.081), breakfast (β = −0.046, *p* = 0.029, 95% CI = −0.087 to −0.005) and physical activity (β = 0.035, *p* = 0.039, 95% CI = 0.002 to 0.068) with HRQoL were significantly observed in the unadjusted model (Pseudo *R*^2^ = 0.1879).

**Table 2:** Tobit regression analysis between sociodemographic characteristics, lifestyle behaviors and HRQoL.

**Variables**	**HRQoL**							
	**Adjusted**				**Unadjusted**			
	β	***p***	**95% CI**		β	***p***	**95% CI**	
Intercept	1.143	< 0.001^**^	1.067	1.219	1.047	< 0.001^**^	0.998	1.096
**Daily routine (ref**. **=** **Irregular)**								
Regular	0.025	0.018^*^	0.004	0.045	0.019	0.101	−0.004	0.041
**Sleep quality (ref**. **=** **Not good)**								
Good	0.049	< 0.001^**^	0.029	0.069	0.059	< 0.001^**^	0.037	0.081
**Breakfast (ref**. **≤4 times/week)**								
≥4 times/week	−0.041	0.036^*^	−0.079	−0.003	−0.046	0.029^*^	−0.087	−0.005
**Smoking (ref**. **=** **No)**								
Yes	−0.027	0.043^*^	−0.053	−0.001	−0.016	0.238	−0.042	0.010
**Drinking (ref**. **=** **No)**								
Yes	−0.009	0.378	−0.031	0.012	−0.011	0.329	−0.033	0.011
**Physical activity (ref**. **=** **No)**								
Yes	0.025	0.094	−0.004	0.055	0.035	0.039^*^	0.002	0.068
**Sex (ref**. **=** **Male)**								
Female	−0.029	0.014^*^	−0.052	−0.006				
**Age (ref**. **≤45)**								
45 ~ 59	−0.004	0.726	−0.026	0.018				
≥60	−0.009	0.759	−0.065	0.047				
**Marital status (ref**. **=** **Single)**								
Married	−0.021	0.504	−0.082	0.040				
Others (e.g., Divorced)	−0.030	0.566	−0.133	0.073				
**Number of children (ref**. **=** **0)**								
1 ~ 2	0.012	0.671	−0.043	0.066				
>2	0.028	0.331	−0.028	0.084				
**Annual household income (ref**. ** ≤ ¥80,000)**								
¥80,000 ~¥1,50,000	−0.052	0.001^**^	−0.083	−0.021				
>¥1,50,000	−0.050	0.002^**^	−0.082	−0.019				
**Education (ref**. **=** **Below undergraduate)**								
Undergraduate	0.002	0.845	−0.020	0.025				
Above undergraduate	0.004	0.820	−0.031	0.039				
**Title (ref**. **=** **Below middle)**								
Middle	−0.004	0.722	−0.027	0.018				
Above middle	−0.021	0.232	−0.054	0.013				
**Enrolment (ref**. **=** **Contract)**								
Permanent	0.019	0.057	−0.001	0.040				
**Chronic diseases (ref**. **=** **No)**								
Yes	−0.118	< 0.001^**^	−0.146	−0.090				
**BMI (ref**. **≤18.5)**								
18.5 ~ 25	−0.016	0.464	−0.058	0.027				
≥25	−0.042	0.081	−0.090	0.005				
**Caregiving status (ref**. **=** **No need)**								
Care for the elderly	0.006	0.565	−0.015	0.027				
**Type of residence (ref**. **=** **Urban)**								
Rural	−0.028	0.011^*^	−0.049	−0.006				
**Commercial insurance (ref**. **=** **No)**								
Yes	−0.004	0.687	−0.024	0.016				

* p < 0.05 and

** p < 0.01, respectively.

## 4. Discussion

The purpose of this study was to explore the relationship between lifestyle behaviors and HRQoL among Chinese primary health care physicians. Our results confirmed the significant association between daily routine, sleep quality, breakfast, smoking and HRQoL, while drinking and physical activity were not found to influence HRQoL of Chinese primary health care physicians.

We found that regular daily routine and good sleep quality were positively associated with HRQoL among Chinese primary health care physicians. Given the 24-h nature of medical care, shift work is common in the healthcare sectors, including evening, night, and early morning shifts, as well as fixed or rotating schedules (56). As a main cause to irregular daily routine, shift work appears to be a risk factor for overweight, obesity, type 2 diabetes, elevated blood pressure, sleep deprivation and the metabolic syndrome, all of which have a poor impact on HRQoL (57, 58). Workers during irregular daily routine will experience the negative influence of sleeping, waking, and eating at an inappropriate circadian phase (59). There is evidence that the circadian rhythms of individuals during an irregular daily routine may deteriorate mood and performance in the healthcare sectors (60, 61). This situation highlights the necessity for examination and intervention programs on negative health outcomes connected with shift work. According to a meta-analysis (8), the pooled prevalence of sleep disturbances among Chinese healthcare professionals was much higher than the general population in China, owing to the changes of lifestyle, increased work pressure, and deteriorating doctor-patient relationships. In addition, physicians are reported more caffeine consumption than the general population (62), which have several negative effects on sleep quality and quantity (63). Poor sleep quality, however, may have a negative impact on immune system and be related to depression and anxiety, which can affect both physical and mental health in HRQoL (64, 65) and result in high levels of burnout causing more medical errors (62, 66, 67). It is suggested that health care policy makers should implement measure to improve sleep quality of primary health care physicians, such as promoting physical activity, strategic naps, work hour reductions and environmental modifications in the workplace (56, 62). Maybe health care organizations can follow an example of some companies which provide rooms with nap pods or beds for the purpose of napping (68).

Our finding that smoking was a risky factor for HRQoL was in accord with earlier researches (45, 46). Physicians may be particularly at risk of smoking, due to heavy workload, work conditions, or nightshifts disrupting the circadian rhythm (69). The overall smoking prevalence among Chinese physicians ranged from 14 to 64% across studies and smoking rates of PHC physicians was 42% (70). On the one hand, smokers are more likely than non-smokers to develop cardio vascular disease, stroke, lung cancer and have higher risks of heart failure (71, 72). On the other hand, physicians who smoke have less knowledge and less favorable attitude toward tobacco control compared to non-smokers (73, 74). Consequently, they would provide less smoking cessation counseling for their patients (75). Furthermore, smoking may cause diminished overall health and increased absenteeism from work (76), which not only have negative impact on the delivery of the healthcare services to their patients but also cause substantial economic burden of sickness absenteeism (77). As a result, it is recommended to develop tailored smoking cessation training programs for primary health care physicians.

Interestingly, we discovered that the frequency of breakfast was inversely connected to HRQoL among primary health care physicians in the current study, whereas a study conducted in Taiwan (78) showed that breakfast skippers had significantly worse HRQoL than breakfast eaters. One possible explanation for this might be that participants are more inclined to skip breakfast for longer sleep durations, since sleep quality (β = 0.049) appears to have greater impact than breakfast (β = −0.042) on HRQoL. Although there are some evidence showed that skipping breakfast is negatively associated with obesity (78), diabetes mellitus (79) and dyslipidemia, the importance of breakfast remains controversial (80, 81). Breakfast skippers seemed to have lower risk for of chronic disease and skipping breakfast as a way for calorie restriction may have potential metabolic benefits including neuroprotective, anti-aging, and anti-inflammatory (81). In addition, there is a study indicating that breakfast skippers showed better HRQOL and lower levels of stress and depression than breakfast eaters who ate a poor or very poor quality of breakfast (40). According to the study, a good quality breakfast comprises of bread/toast/cereal and/or dairy products rather than commercially prepared goods. Moreover, compared with a nutrition-inadequate breakfast and no breakfast, a nutrition-adequate breakfast will significantly improve short-term cognitive function (82), emphasizing the importance of breakfast quality. Physicians may have limited access to healthy breakfast due to work commitments and lack of time in the workplace (83). Hence, the association between breakfast quality and HRQoL should be further studied in future.

Furthermore, an increasing number of studies have been conducted to examine the relationship between physical activity and HRQoL (47, 84, 85). It was evidently demonstrated that physical activity improved HRQoL and well-being when compared with minimal or no-treatment controls for adults aged 18–65 years (86). The typical primary health care physician works long hours and leads a sedentary lifestyle, thus they are encouraged to shift from being sedentary to doing some physical activity which has the greatest potential health gains (87). However, contrary to expectations, physical activity was not significantly associated with HRQoL based on our regression result and this finding was in line with a research among PHC physician in Saudi Arabia (38). Nonetheless, Mann–Whitney U analysis showed that participants who had a habit of physical activity (90.94%) got significantly better HRQoL. The insignificant regression results may be due to the fact that most of the participants had a habit of physical activity, which requires further research on a larger scale. Also, a recent study discovered that shift work seemed to hinder the beneficial effects of physical activity (88). Similarly to physical activity, drinking was not found significantly associated with HRQoL in line with some researches (39, 89) while some studies have demonstrated that drinking is a risk factor for HRQoL (46, 47, 90). By contrast, some studies have reported moderate drinkers tending to have better HRQoL than non-drinkers and heavy drinkers (16, 91, 92). Besides, drinking was found to effectively alleviate stress and be linked to improved mental health in healthcare professionals (93, 94). Such the existence of a causal relationship between alcohol consumption patterns and HRQoL may be resulted from differences in consumption by sex, nationality, and individual characteristics (92). Moreover, a review indicated that alcohol consumption was an independent predictor of chronic conditions (95), which was a significantly negative factor of HRQoL in our study. Our result also could be partly explained by the inclusion of participants who refrain from alcohol consumption permanently or temporarily due to the presence of chronic disease, which consequently would decrease their HRQoL. Therefore, further studies including more specific drinking groups should be conducted in the future to determine the precise correlations between alcohol consumption and HRQoL in primary health care physicians.

Approximately 18.1% of the study participants reported problems in the Anxiety/Depression dimension which was higher than the other four dimensions. Many studies indicated that physicians can be affected by the full spectrum of mental disorders and the most common mental disorders reported among physicians are depression and anxiety (96–98). Workplace risk factors, such as high job demands, a work-family life imbalance and long working hours, are often important in explaining much of the variation in mental ill health among physicians (99). Working as a primary health care physician involves a heavy workload, and consequently they have little leisure time to spend with their family and friends. Furthermore, healthcare workers are always at the frontline of public health responses to major critical incidents and emergencies like COVID-19 (100, 101), causing increases in mental ill health among them. In this regard, it is high time that public health care policy makers should establish ways to mitigate mental health risks and tailor interventions, such as modifications to work processes, shortening of shifts (98) and mental health training, which effectively make an enhancement in some mental health outcomes among primary health care physicians. Another potential solution is eHealth interventions including Cognitive Behavioral Therapy, Stress Management, Mindfulness approaches and Cognitive training through Apps, which appears to be an effective and more feasible way than face-to-face sessions to deliver these types of interventions among PHC physicians (102, 103).

Several study limitations are worth mentioning. First, our results should be generalized with caution to other countries with different cultural backgrounds because this was a cross-sectional study in China. Second, physicians also might have reported underestimation of unfavorable lifestyle behaviors or overestimation of HRQoL in this study because of social acceptability bias. Third, dietary behaviors were not included. It should be fully studied in future. Lastly, we used a simple questionnaire of lifestyle behaviors for greater response by participants, which might result in missing important information. We suggest that future studies use standardized questionnaires to comprehensively measure daily routine, sleep quality, breakfast, smoking, drinking and physical activity. For example, physical activity can be assessed by International Physical Activity Questionnaire (IPAQ) (104).

## 5. Conclusions

This study evaluated association of lifestyle behaviors including daily routine, sleep quality, frequency of eating breakfast, physical activity, drinking, and smoking with HRQoL in a group of 894 Chinese primary health care physicians. Our study found that lifestyle behaviors including daily routine, sleep quality, frequency of eating breakfast, and smoking affected the HRQoL of Chinese primary health care physicians. However, this association was not observed for other lifestyle behaviors including drinking and physical activity. These findings may guide health policy makers to tailor interventions, such as adequate sleep, strategic naps, and smoking cessation training programs, to improve HRQoL of primary health care physicians effectively.

## Data availability statement

The raw data supporting the conclusions of this article will be made available by the authors, without undue reservation.

## Ethics statement

The research design was reviewed and approved by the Ethics Committee of China Pharmaceutical University, Nanjing city, Jiangsu Province, China (Project Number: CPU2014006). All methods were carried out in accordance with the relevant guidelines and regulations. Based on the principle of informed consent, all data were collected anonymously after obtaining the permission and informed consent signed by respondents.

## Author contributions

YH and XX contributed to the conception and design of the study. YL and YH contributed to the data analysis. YL, YH, and XX contributed to the interpretation of data. YL contributed to writing manuscript. All authors had read and approved the final version of the manuscript for submission.
