# Supplementary material for: Association between lifestyle behaviors and health-related quality of life among primary health care physicians in China: A cross-sectional study
Source: Front Public Health. 2023 Mar 8;11:1131031. doi: 10.3389/fpubh.2023.1131031 (PMC10030863; doi:10.3389/fpubh.2023.1131031)
Supplement: Supplementary file 1 [file Table_1.DOCX]

Supplementary Material

Association Between Lifestyle Behaviors and Health-Related Quality of Life Among Primary Health Care Physicians in China: A Cross-Sectional Study

Yisha Lin, Yuankai Huang, Xiaoyu Xi*

*** Correspondence:** Xiaoyu Xi: xixy@cpu.edu.cn

# Supplementary Table

Appendix 1: Robustness checks

**Table 1 Robust test for Tobit regression analysis between sociodemographic characteristics, lifestyle behaviors and HRQoL**

| Variables | HRQoL | | | | |
| --- | --- | --- | --- | --- | --- |
|  | **Coefficient** | ***p* Value** | **95% CI** | | |
| Intercept | 0.902 | <0.001** | 0.847 | 0.957 | |
| Daily routine (ref.= Irregular) | | | | | |
| Regular | 0.018 | 0.015 | 0.004 | 0.033 | |
| Sleep quality (ref.= Not good) | | | | | |
| Good | 0.036 | <0.001** | 0.021 | 0.050 | |
| Breakfast (ref.= < 4 times/week) | | | | | |
| ≥ 4 times/week | -0.030 | 0.034* | -0.057 | -0.002 | |
| Smoking (ref.= No) | | | | | |
| Yes | -0.020 | 0.041* | -0.038 | -0.001 | |
| Drinking (ref.= No) | | | | | |
| Yes | -0.007 | 0.400 | -0.022 | 0.009 | |
| Physical activity (ref.= No) | | | | | |
| Yes | 0.018 | 0.100 | -0.003 | 0.039 | |
| Sex (ref.= Male) | | | | | |
| Female | -0.021 | 0.015* | -0.037 | -0.004 | |
| Age (ref.= < 45) | | | | | |
| 45 ~ 59 | -0.002 | 0.764 | -0.018 | 0.014 | |
| ≥ 60 | -0.007 | 0.743 | -0.047 | 0.034 | |
| Marital status (ref.= Single) | | | | | |
| Married | -0.016 | 0.488 | -0.060 | | 0.029 |
| Others (e.g., Divorced) | -0.023 | 0.536 | -0.098 | 0.051 | |
| Number of children (ref.= 0) | | | | | |
| 1 ~ 2 | 0.009 | 0.661 | -0.031 | 0.048 | |
| > 2 | 0.020 | 0.323 | -0.020 | 0.061 | |
| Annual household income (ref.= <￥80000) | | | | | |
| ￥80000 ~￥150000 | -0.038 | 0.001** | -0.060 | -0.015 | |
| >￥150000 | -0.036 | 0.002** | -0.059 | -0.013 | |
| Education (ref.= Below undergraduate) | | | | | |
| Undergraduate | 0.001 | 0.894 | -0.015 | 0.018 | |
| Above undergraduate | 0.002 | 0.887 | -0.024 | 0.027 | |
| Title (ref.= Below middle) | | | | | |
| Middle | -0.003 | 0.745 | -0.019 | 0.014 | |
| Above middle | -0.014 | 0.256 | -0.039 | 0.010 | |
| Enrolment (ref.= Contract) | | | | | |
| Permanent | 0.015 | 0.049* | 0.000 | 0.029 | |
| Chronic diseases (ref.= No) | | | | | |
| Yes | -0.084 | <0.001** | -0.104 | -0.063 | |
| BMI (ref.= < 18.5) | | | | | |
| 18.5 ~ 25 | -0.011 | 0.478 | -0.042 | 0.020 | |
| ≥ 25 | -0.030 | 0.091 | -0.064 | 0.005 | |
| Caregiving status (ref.= No need) | | | | | |
| Care for the elderly | 0.004 | 0.596 | -0.011 | 0.019 | |
| Type of residence (ref.= Urban) | | | | | |
| Rural | -0.020 | 0.009** | -0.036 | -0.005 | |
| Commercial insurance (ref.= No) | | | | | |
| Yes | -0.004 | 0.628 | -0.018 | 0.011 | |
